# HIV-related stress predicts depression over five years among people living with HIV

**DOI:** 10.3389/fpubh.2023.1163604

**Published:** 2023-06-12

**Authors:** Zongyan Liu, Xi Chen, Jie Li, Zhi Xie, Yunxiang Huang, Dan Luo

**Affiliations:** ^1^Xiangya School of Public Health, Central South University, Changsha, China; ^2^Hunan Provincial Center for Disease Control and Prevention, Changsha, Hunan, China; ^3^Furong District Center for Disease Control and Prevention, Changsha, Hunan, China; ^4^Changsha Center for Disease Control and Prevention, Changsha, Hunan, China

**Keywords:** cohort studies, depression, PLWH, HIV-related stress, social support

## Abstract

**Introduction:**

Extant literature has demonstrated significant associations between HIV-related stress, social support, and depression among PLWH. However, little research has been conducted on the changes in such associations over time. Our study aims to explore the longitudinal relationship between HIV-related stress, social support, and depression among PLWH over five years.

**Methods:**

320 PLWH were recruited from Changsha Center for Disease Control and Prevention (CDC), Hunan Province, China. They were assessed for depressive symptoms, HIV-related stress, and social support within 1 month of HIV diagnosis, 1 year after diagnosis, and five years after diagnosis, respectively. Relationships between these variables were examined using a fixed effect model.

**Result:**

The prevalence of depressive symptoms within the first month, first year, and fifth years of HIV diagnosis was 35, 12.2, and 14.7%, respectively. Emotional stress (*β*: 0.730, 95% CI: 0.648, 0.811), social stress (*β*: 0.066, 95% CI: 0.010, 0.123), instrumental stress (*β*: 0.133, 95% CI:0.046, 0.221) positively predicted depression, while social support utilization (*β*: −0.176, 95% CI: −0.303, −0.049) negatively predicted depression.

**Conclusion:**

Our study suggests that HIV-related stress and social support predict depressive symptoms over time among PLWH and that reducing HIV-related stress and improving social support in the early stages of diagnosis is extremely important in preventing depressive symptoms among PLWH.

## 1. Introduction

HIV infection is a major global health burden, with 38.4 million people infected and 650 thousand people dying of AIDS-related illnesses in 2021 (1). People Living with HIV (PLWH) are faced with multiple challenges including social stigma, social isolation, and worry about the future, which predisposes them to an increased risk of mental disorder (2). Depression is the most common mental disorder among PLWH, with a meta-analysis showing the prevalence of depression in PLWH was two times higher than in the general population (3). Another meta-analysis reported that the pooled prevalence of depression was 39% among PLWH, with a range of 12.8–78% by various studies in various countries (4). A meta-analysis by Wang et al. showed that the pooled prevalence of depression or depressive symptoms among PLWH in China was 50.8% (5). Depression is characterized by a wide range of symptoms that affect an individual’s daily life, including low mood, persistent sadness, poor concentration, pessimistic thoughts, social withdrawal, and loss of interest in the things people usually enjoy (6). It is one of the leading causes of morbidity and mortality among PLWH (7) and is associated with decreased immunity function, severe disease progression, poorer treatment adherence, lower quality of life, and shorter life expectancy (8).

In light of the high prevalence of depression and its significant negative health outcomes among PLWH, it is crucial to identify influencing factors of depression to guide further interventions. Among the various factors that affect depression among PLWH, HIV-related stress is one of the most well-established ones. Being infected with HIV is an extremely stressful experience that may affect every aspect of life (9, 10). Concerns about AIDS-related clinical symptoms, drug side effects, AIDS-related social stigma, and uncertainty about disease outcomes all bring enormous stress to PLWH (10–12). Studies have shown PLWH under great stress were at an increased risk of health-damaging behaviors such as smoking (13), alcohol consumption (14), and unsafe sex (15), which may lead to depression. A higher level of HIV-related stress has been shown to be associated with a higher prevalence of depression in PLWH (16), which may be explained by the disturbances in 5-hydroxytryptamine and stress hormones caused by persistent stress (17).

Social support is another well-established factor that affects depression among PLWH. A recent meta-analysis showed that PLWHs with poor social support were over two times more likely to develop depression than those with strong social support (18). Adequate social support is helpful to relieve HIV-related stress, improve physical and mental health, and reduce the prevalence of depression (16). In addition, previous studies in our research group have shown that adequate social support can reduce HIV-related stress levels in PLWH (19). Two general models have been proposed to elucidate the beneficial role of social support on depression: the direct effect model and the stress buffer model. In the direct effect model, social support has a direct positive effect on preventing depression (20). In the stress buffer model, social support can mitigate the negative effects of negative events on depression (21). A large body of evidence has demonstrated that social support decreases the level of depression among PLWH either directly, or indirectly through its buffering effect (21, 22). The relationship among HIV-related stress, social support, and depression among PLWH may be explained by the conceptual framework of stress, social support, and health behavior to understand the multiple determinants of health status, in this case, depression or depressive symptoms (23, 24). According to this model, PLWH’s depression is affected by HIV-related stress and several protective factors (e.g., social support) have both direct and indirect moderating effects on depression.

Although abundant evidence has shown the positive association between HIV-related stress and depression as well as the negative association between social support and depression, such associations are mainly based on cross-sectional investigations. Longitudinal studies allow the exploration of time effects and patterns of relationships over time. To the best of our knowledge, however, little research has been conducted on how HIV-related stress and social support influence depression over time. Therefore, our study aims to explore the longitudinal relationship between HIV-related stress, social support, and depression among PLWH over five years.

## 2. Methods

### 2.1. Study design and participants

Based on the support and cooperation of Changsha CDC professionals, the five-year longitudinal observational study was conducted at the Changsha Center for Disease Control and Prevention (CDC), Hunan Province, China. Changsha City, located in the south-central region of China, is the capital city of Hunan Province, China. A previous study reported a total of 3,624 cases of PLWH in Changsha City between 2011 and 2016, including 3,145 males and 479 females (25). We conducted the baseline survey from March 2013 to September 2014, the first (one-year) follow-up survey from March 2014 to October 2015, and the second (five-year) follow-up survey from March 2018 to April 2019. To be eligible for participation individuals had to be 18 years or older, receiving an HIV diagnosis ≤1 month, and living in Changsha for at least 6 months. The reason we included newly diagnosed PLWH only was to better examine the effect of time effects on depression since the impact of HIV infection diagnosis may change over time and the risk of depression among PLWH may be different at different time points of HIV infection diagnosis. Participants were excluded if they were unable to understand the study procedures due to illiteracy, intoxication, or cognitive difficulties observed by an interviewer.

All study procedures were approved by the Ethics Committee of Central South University. Before the formal investigation, the investigators explained in detail the study’s purpose, content, and significance to participants. Participants were also informed that their personal information would be kept confidential. After providing written informed consent, participants were invited to complete the questionnaires through face-to-face or telephone interviews.

**Figure 1:**
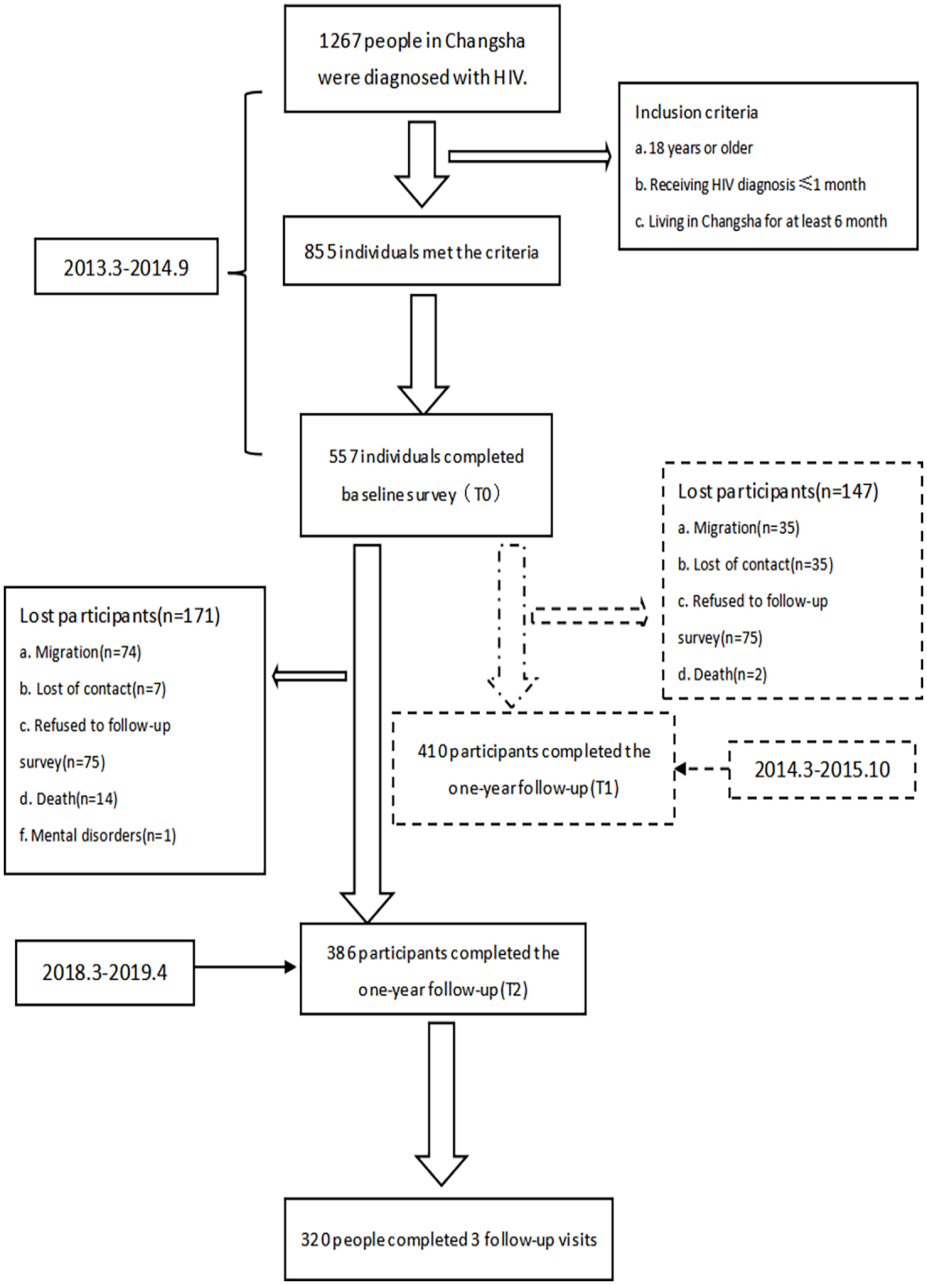
Flowchart of participant enrollment.

During the baseline survey, 1,267 PLWH were newly diagnosed in Changsha CDC, of whom 855 met the inclusion criteria, and 557 eventually participated in the baseline survey. Among the 557 participants who completed the baseline survey, 410 completed the first follow-up survey, and 386 completed the second follow-up survey. Finally, 320 participants completed all three surveys. Figure 1 shows a detailed flow chart of participant recruitment.

### 2.2. Measures

#### 2.2.1. Background information

Social demographic information was collected through a self-administered background questionnaire, which included gender, age, residence, sexual orientation, marital status, education level, and work status. After the study team signed the information confidentiality agreement and obtained the permission of the local CDC, participants’ HIV-related clinical information (CD4 count and whether they were receiving antiretroviral therapy) was obtained through the Chinese HIV/AIDS Comprehensive Response Information Management System (CRIMS), which run by CDC.

#### 2.2.2. Depression

The 9-item Patient Health Questionnaire (PHQ-9) was used to assess depressive symptoms in study subjects over the past 2 weeks. Each item is scored on a four-point Likert scale from 0 (not at all) to 3 (nearly every day). The total score ranges from 0–27, with higher scores indicating more depressive symptom (26). The PHQ-9 showed good internal consistency in our baseline survey, with a Cronbach’s α coefficient of 0.914.

#### 2.2.3. Social support

The 10-item Social Support Rating Scale (SSRS) was used to assess perceived social support from three domains: objective support (3 items), subjective support (4 items), and support utilization (3 items) (27). Subjective support reflects the patient’s subjective emotional experience and satisfaction of being respected, supported, or understood. Objective support reflects the support the patient believes he or she receives, including both direct assistance and social associations. Social support utilization reflects the extent to which the patient makes use of social support. The total score ranges from 12–66, with higher scores indicating higher levels of social support. The SSRS showed good internal consistency in our baseline survey, with a Cronbach’s α coefficient of 0.805.

#### 2.2.4. HIV-related stress

HIV-related stress was assessed by the 17-item Chinese HIV/AIDS Stress Scale (CSS-HIV), which is an AIDS-specific stress scale and assesses stress levels from three dimensions: emotional stress (7 items), social stress (10 items), and instrumental stress (6 items) (28). Emotional stress includes HIV/AIDS-related grief/bereavement, distressing emotions, and concerns about death. Social stress includes stressful social events such as isolation, stigma, difficulties in disclosure of HIV status, and interpersonal associations. Instrumental stress includes daily practical difficulties associated with HIV/AIDS-related financial, transport, and treatment problems. Each item is rated on a 5-point Likert scale from 0 (not at all) to 4 (extremely). The total score ranges from 0–68, with higher scores indicating higher levels of HIV-related stress. The CSS-HIV showed good internal consistency in our baseline survey, with a Cronbach’s α coefficient of 0.903.

### 2.3. Statistical analysis

To compare whether there were differences in baseline characteristics between those who completed three follow-up visits and those who did not, chi-square tests were used for categorical variables, and two independent samples t-tests were used for quantitative data. ANOVA was used to compare the scores of social support, HIV-related stress, and CD4 counts at the three time points. Chi-square tests were used to compare the prevalence of depression and treatment rate at the three time points. Finally, the correlation between HIV-related stress, social support, and depression, as well as the interaction between HIV-related stress, social support, and time were analyzed separately by a mixed-effects model. A mixed effects model is a statistical test used to predict a single variable using two or more other variables and is most widely used in a longitudinal study where individuals are followed over a period of time and data are collected at multiple time points (29). A mixed effects model uses both fixed and random effects to reflect a hierarchy of levels with repeated, correlated measurements among all levels (29). The fixed effects show the population mean differences between any set of treatments while the random effects represent the general variability among subjects or units (29).

## 3. Results

### 3.1. Characteristics of participants

Table 1 shows the baseline characteristics of the participants. Among the 320 participants who completed all three visits, slightly over half were of urban residence (50.9%), aged≥30 (53.7%), single (58.4%), and with high school and below education (54.2%). Most were males (90.9%) and non-heterosexual (61.87%). 112 participants screened positive for depression (35%). The mean values of HIV-related stress, social support, and CD4 count were 22.61, 28.91, and 367.13, respectively.

**Table 1:** Baseline characteristics of the participants who completed all three visits and those who did not.

Characteristics	Completed all three visits (*n* = 320) *n* (%)	Lost to follow-up (*n* = 237) *n* (%)	*p*
Residence			0.943
Rural	157 (49.1)	117 (49.4)	
Urban	163 (50.9)	120 (50.6)	
Age			0.121
<30	172 (53.7)	143 (60.3)	
≥30	148 (46.3)	94 (39.7)	
Sex			0.004
Male	291 (90.9)	224 (94.5)	
Female	29 (9.1)	13(5.5)	
Marital status			0.011
Single	187 (58.4)	160 (67.5)	
Married	95 (29.7)	44 (18.6)	
Divorced/widowed	38 (11.9)	33 (13.9)	
Education			0.537
High school and below	139 (43.4)	122 (51.8)	
College and above	181 (56.6)	115 (48.5)	
Sexual orientation			0.542
Heterosexuality	122 (38.1)	81 (34.2)	
Homosexuality	129 (40.3)	106 (44.7)	
Bisexual	69 (21.6)	50 (21.1)	
Depression			0.941
yes	112 (35.0)	84 (35.4)	
no	208 (65.0)	153 (64.6)	
HIV-related stress, mean (SD)	22.61 (13.28)	22.88 (13.24)	0.812
Social support,mean, (SD)	28.91 (7.80)	29.31 (9.06)	0.567
CD4 count, cells/mm3, mean (SD)	365.92 (187.67)	379.58 (184.45)	0.522

A comparison of the sample characteristics between those who completed all three visits and those who did not (lost to follow-up) showed no significant differences in all characteristics except for gender and marital status. Compared to those who completed all three surveys, those who were lost to follow-up were more likely to be males (94.5% vs. 90.9, *p* = 0.004) and single (67.5% vs. 58.4%, *p* = 0.011).

### 3.2. Changes in psychosocial characteristics during three visits

**Figure 2:**
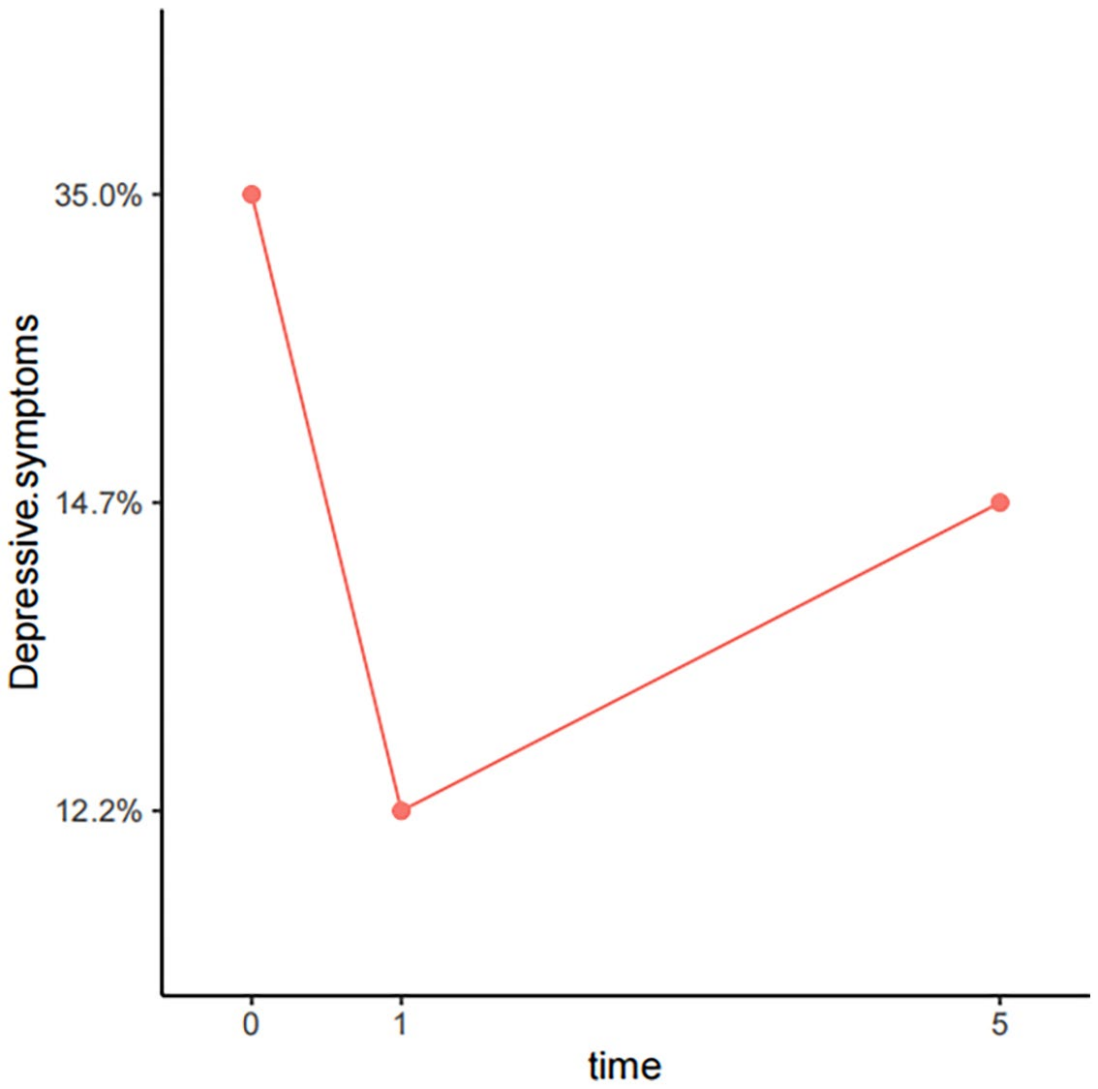
Five-year trajectory chart of depressive symptoms.

**Figure 3:**
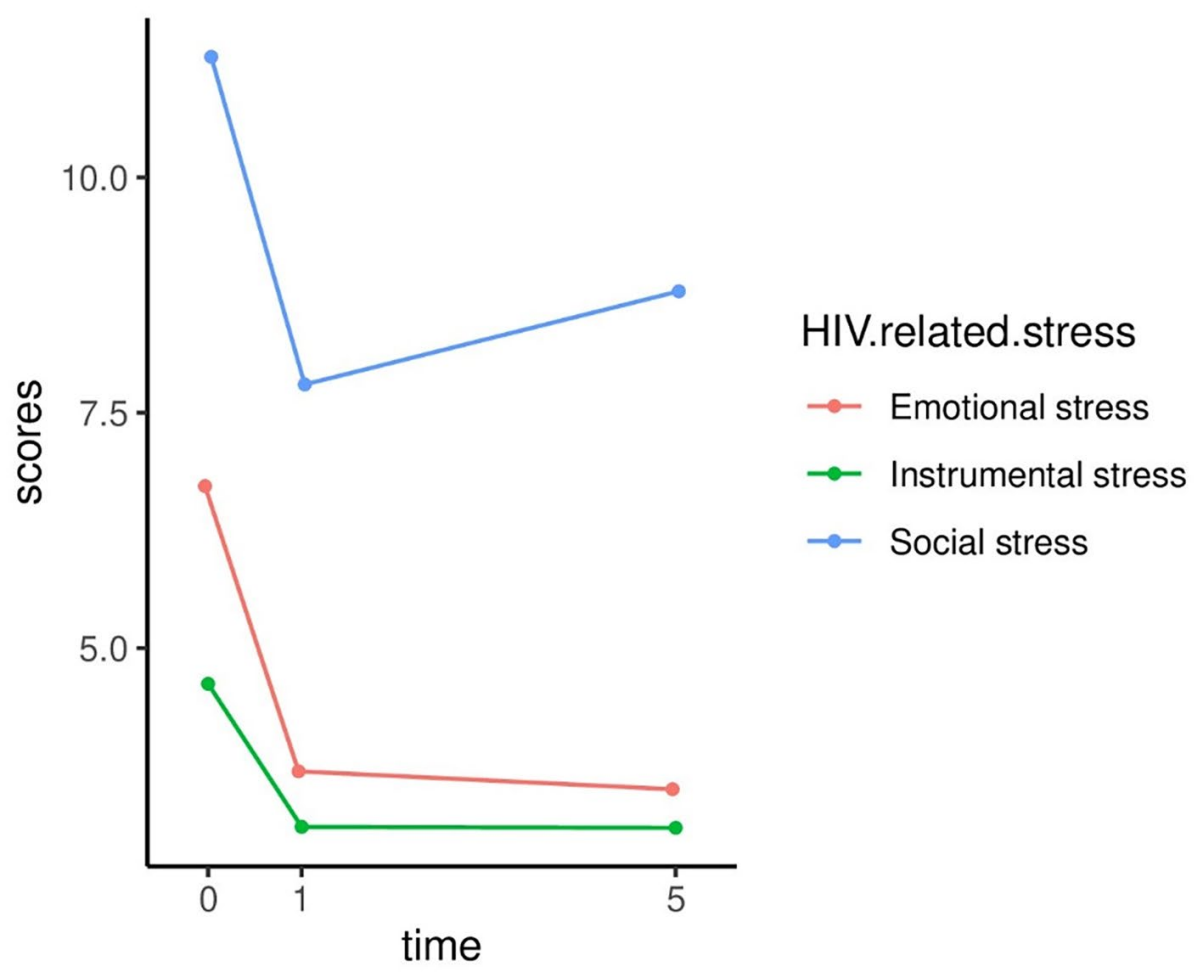
Five-year trajectory chart of HIV-related stress.

**Figure 4:**
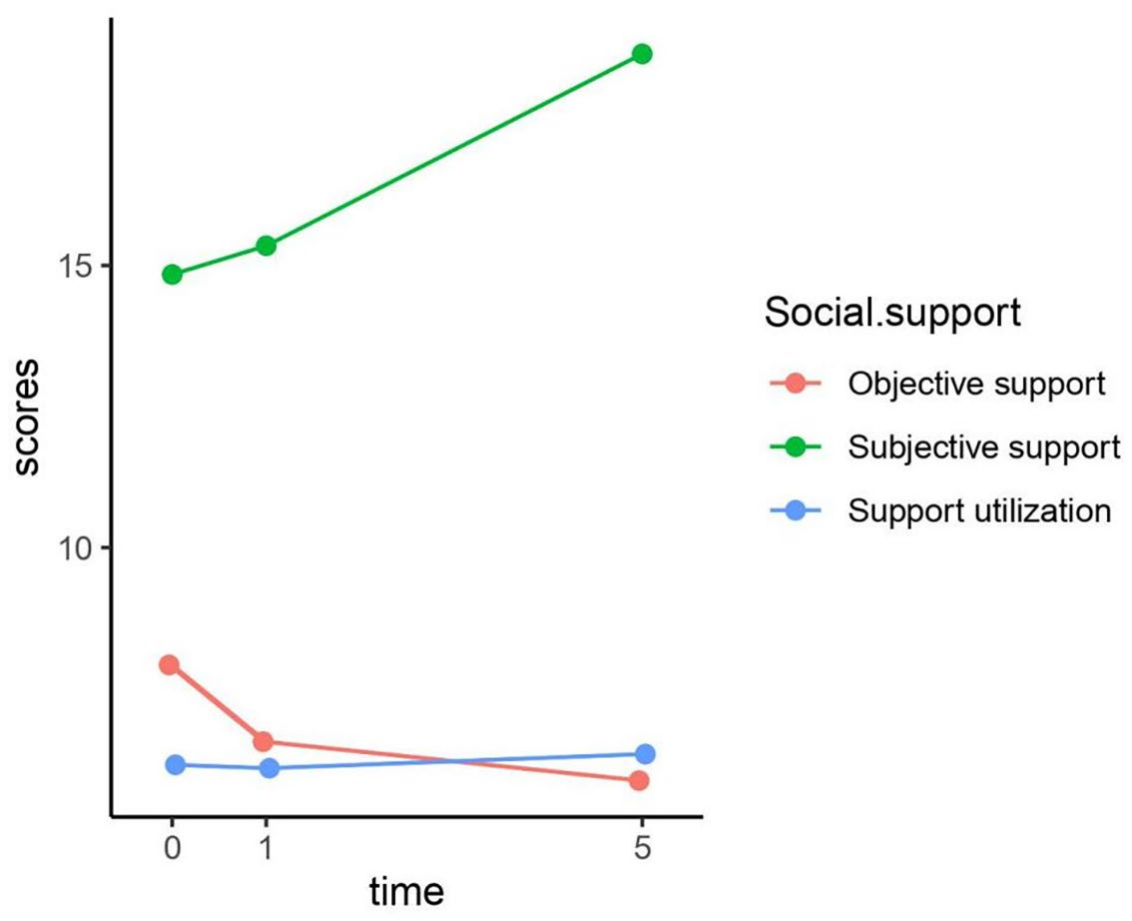
Five-year trajectory chart of social support.

Table 2, Figures 2–4 show the changes in depression prevalence, HIV-related stress, and social support during the five years. The prevalence of depression was 35% at baseline, declined to 12.2% at the first-year follow-up, and then increased slightly to 14.7% at the fifth-year follow-up, showing significant differences over time (*p* < 0.001). All three dimensions of HIV-related stress showed significant differences in three visits, with a generally declining trend over time (Figure 3). For social support, both subjective and objective support showed significant differences in three visits, with a declining trend over time. However, support utilization showed no significant difference during the three visits (Figure 4).

**Table 2:** Changes of psychosocial characteristics.

Characteristics	T0	T1	T2	*p*
Depressive symptoms				<0.001
yes (%)	112 (35)	39 (12.2)	47 (14.7)	
no (%)	208 (65)	281 (87.8)	273 (85.3)	
HIV-related stress				
Emotional stress, mean (SD)	6.72 (5.19)	3.69 (3.85)	3.50 (4.55)	<0.001
Social stress, mean (SD)	11.28 (5.75)	7.80 (5.82)	8.79 (5.50)	<0.001
Instrumental stress, mean (SD)	4.62 (4.24)	3.10 (3.54)	3.09(3.94)	<0.001
Social support				
Subjective support, mean (SD)	14.84 (5.51)	15.35 (6.65)	18.75 (4.70)	<0.001
Objective support, mean (SD)	7.92 (9.91)	6.56 (2.79)	5.87 (2.71)	<0.001
Support utilization, mean (SD)	6.15 (1.75)	6.09 (1.91)	6.34 (2.44)	0.154

The prevalence of depressive symptom is tested using chi-square tests, and other variables were analyzed using ANOVA. *p* < 0.05 indicated significant differences between variables at different time points.

### 3.3. The association between social support, HIV-related stress, and depression

Table 3 shows the association between social support, HIV-related stress, and depression using a mixed-effects model. We chose a linear model as the linking function to analyze the fixed effects of all variables. In model 1, after adjusting for sample characteristics, HIV-related stress showed a significant positive association with depression (β: 0.306, 95% CI: 0.284, 0.327). In model 5, after adjusting for sample characteristics, social support showed a significant negative association with depression (β: -0.158, 95% CI: −0.201, −0.115). There was no significant interaction between HIV-related stress and social support.

**Table 3:** Association between HIV-related stress, social support with depression.

Characteristics	Model 1β (95%CI)	*p*	Model 2β (95%CI)	*p*	Model 3β (95%CI)	*p*
Time						
T0	1.688 (0.957，2.420)	<0.001	3.557 (2.614，4.500)	<0.001	1.656 (0.928，2.383)	<0.001
T1	0.612 (0.003，1.222)	0.0049	−0.133 (−0.949, −0.684)	0.750	0.449 (−0.164，1.062)	0.151
T2						
HIV-related stress	0.306 (0.284，0.327)	<0.001			0.308 (0.237，0.379)	<0.001
Social support			−0.158 (−0.201, −0.115)	<0.001	−0.048 (−0.100，0.004)	0.068
HIV-related stress × Social support					<0.001 (−0.002，0.001)	0.612

Model 1: Number of measurements + HIV-related stress after adjusting for socio-demographic and clinical characteristics; Model 2: Number of measurements + social support after adjusting for socio-demographic and clinical characteristics; Model 3: Number of measurements + HIV-related stress + social support + interaction of HIV-related stress with Social support after adjusting for socio-demographic and clinical characteristics.

Table 4 shows the association between three dimensions of social support, three dimensions of HIV-related stress, and depression using a mixed-effects model. We chose a linear model as the linking function to analyze the fixed effects of all variables. In model 4, after adjusting for sample characteristics, all three dimensions of HIV-related stress showed significant positive associations with depression, with β ranging from 0.067 to 0.751 (95% CI: 0.011, 0.832). In model 5, after adjusting for sample characteristics, all three dimensions of social support showed significant negative associations with depression, with β ranging from −0.086 to −0.461 (95% CI: −0.641, −0.017). In model 6, after adjusting for sample characteristics and adding social support, the positive effects of emotional stress, social stress, and instrumental stress on PLWH depression were still significant (β: 0.066–0.730, 95% CI:0.010, 0.811), while only support utilization showed a significant negative association with depression (β: -0.176, 95% CI: −0.303, −0.049).

**Table 4:** Relationship between the three dimensions of HIV-related stress, the three dimensions of social support, and depression.

Characteristics	Model 4β (95%CI)	*p*	Model 5β (95%CI)	*p*	Model 6β (95%CI)	*p*
Time						
T0	1.241 (0.387，2.095)	0.004	3.587 (2.377，4.796)	<0.001	1.248 (0.371，2.125)	0.005
T1	0.337 (−0.267, 0.942)	0.274	0.117 (−0.783, 1.016)	0.799	−0.252 (−0.339, 0.843)	0.403
T2						
HIV-related stress						
Emotional stress	0.751 (0.671，0.832)	<0.001			0.730 (0.648，0.811)	<0.001
Social stress	0.067 (0.011，0.123)	0.019			0.066 (0.010，0.123)	0.021
Instrumental stress	0.138 (0.050，0.266)	0.002			0.133 (0.046，0.221)	0.003
Social support						
Subjective support			−0.086 (−0.156, −0.017)	0.015	−0.002 (−0.051, 0.048)	0.952
Objective support			−0.154 (−0.291, −0.018)	0.026	−0.036 (−0.133, 0.061)	0.464
Support utilization			−0.461 (−0.641, −0.281)	<0.001	−0.176 (−0.303, −0.049)	0.007

Model 4: Number of measurements + HIV-related stress after adjusting for socio-demographic and clinical characteristics; Model 5: Number of measurements + social support after adjusting for socio-demographic and clinical characteristics; Model 6: Number of measurements + HIV-related stress + social support after adjusting for socio-demographic and clinical characteristics.

Table 5 shows the interaction between HIV-related stress, social support, and time. We chose a linear model as the linking function to analyze the fixed effects of all variables. For HIV-related stress, two dimensions showed significant interactions with time: emotional stress (*β*: 0.197, 95% CI:0.173,0.221) and instrumental stress (*β*: 0.028, 95%CI:0.002,0.054). For social support, only the subjective support (*β*: -0.042, 95% CI: −0.065, −0.019) and social support utilization (*β*: -0.135, 95% CI: −0.183, −0.088) showed significant interaction with time.

**Table 5:** The interaction of HIV-related stress, social support with time.

Characteristics	Model 7β (95%CI)	*p*	Characteristics	Model 8β (95%CI)	*p*
Time			Time		
T0	6.699 (5.839，7.599)	<0.001	T0	−2.717 (−4.620, −0.813)	0.005
T1	2.894 (2.202，3.585)	<0.001	T1	−4.839 (−6.375, −3.303)	<0.001
T2			T2		
Interaction			Interaction		
Time × Emotional stress	0.197 (0.173，0.221)	<0.001	Time × Subjective support	−0.042 (−0.065, −0.019)	<0.001
Time × Social stress	0.008 (−0.009，0.025)	0.378	Time × Objective support	0.001 (−0.042，0.043)	0.976
Time × Instrumental stress	0.028(0.002，0.054)	0.035	Time × Support utilization	−0.135 (−0.183, −0.088)	<0.001

Model 7: Number of measurements + interaction of HIV-related stress with time after adjusting for socio-demographic and clinical characteristics; Model 8: Number of measurements + interaction of social support with time after adjusting for socio-demographic and clinical characteristics.

In addition, we further run an interaction analysis between support utilization and the three dimensions of stress. Our results showed significant interactions between support utilization and emotional stress (*β*: 0.128, 95% CI: 0.113,0.143), as well as between support utilization and instrumental stress (*β*: 0.024, 95% CI: 0.008, 0.040) (Supplementary Table S1).

## 4. Discussion

Based on a longitudinal study design, our study describes the five-year trajectory of depression, HIV-related stress, and social support among PLWH and explores their associations over time. Our major findings were that both depression and HIV-related stress showed a significant decrease over time, while social support showed a significant increase over time. All three dimensions of HIV-related stress and the support utilization dimension of social support predicted depression over time. Our findings add to the current literature and provide important implications for future interventions to provide more efficient social support and stress management services to PLWH at different stages of their diagnosis.

Our study showed a significant decrease in depression prevalence among PLWH over time, from 35% 1 month after diagnosis to 12.2% 1 year after diagnosis, and 14.7% five years after diagnosis. Over time, the relief of depressive symptoms among newly-diagnosed PLWH may reflect the positive transformation during their struggle with the traumatic event of HIV diagnosis, which has been referred to as post-traumatic growth (PTG) (30–32). There is growing empirical evidence showing that PTG exists among PLWH and helps promote positive mental health growth (30–32). With PTG, PLWH were able to gradually accept their HIV infection over time and were able to cope positively, including actively learning about HIV and seeking help from professional institutions (30–32). This finding suggests that identification and improvement of PTG among PLWH may help foster positive transformation and prevent the occurrence of depression. Furthermore, the significant changes in depression prevalence over time also suggest the assessment and reporting of depression should consider the diagnosis time. However, most of the previous studies did not differentiate the timing of HIV diagnosis when reporting the prevalence of depression (33–36), which may explain the large discrepancy observed in various studies. Our longitudinal study design makes it possible to observe the dynamic changes of depression among PLWH at different stages of the disease and is thus recommended for future research.

Our study showed that HIV-related stress including emotional stress, social stress, and instrumental stress all predicted depression over five years, even after controlling for socio-demographic and clinical characteristics as well as social support. The findings were consistent with previous literature showing HIV-related stressors as a major contributor to depression development among PLWH (37, 38). Our study also showed that emotional stress, social stress, and instrumental stress were highest in PLWH at baseline, presenting a consistent downward trend over the five years. This pattern was consistent with the changes in depression prevalence over the same period, further corroborating the positive associations between HIV-related stress and depression. This finding suggests that prevention of depression among PLWH may benefit from alleviating their HIV-related stress, especially at the initial diagnosis stage when their stress levels were the highest. In addition, the positive interactions between emotional stress, instrumental stress, and time suggest that the effects of emotional and instrumental stress on depression increased over time. This finding indicates the need to start intervention to relieve HIV-related stress as early as possible to reduce its long-lasting negative effects on the later development of depression.

The result indicated that social support including subjective support, objective support, and social support utilization all negatively predicted depression after adjusting for socio-demographic and clinical characteristics. Social support is usually defined as a person’s material or emotional support from others (39). Our finding was consistent with the wide literature showing the protective role of social support in promoting mental health and preventing depression among PLWH (18, 38, 40, 41). However, after further adjusting for the three dimensions of HIV-related stress, only social support utilization showed a significant negative association with depression. Social support utilization represents the degree to which individuals proactively seek help and resources to help them deal with challenges and difficulties (42). Our finding suggests that the actual utilization of social support, rather than the availability of social support plays an essential role in improving mental health and preventing depression among PLWH. This has implications for future depression prevention programs to focus not only on the provision of social support but also the encouragement of PLWH to actively utilize support to achieve the best health-promoting benefits. Furthermore, the negative interactions between subjective support, social support utilization, and time suggest that the effects of subjective support and social support utilization on depression diminish over time. Therefore, providing subjective support and promoting support utilization in the early stage of HIV diagnosis may achieve maximal benefits in depression prevention.

In general, the finding that HIV-related stress and social support predicted depressive symptoms among PLWH supports the application of the conceptual framework of stress, social support, and health behavior among PLWH. This model furthers our understanding of the multiple determinants of PLWH’s depression and highlights the complicated interactions among the multiple determinants. PLWH’s depression is a dynamic process over time and is affected by multiple factors. Our findings provide important insights for future research to investigate the mental health of PLWH under a broader and multilevel background and develop comprehensive intervention programs that involve different components such as a combination of stress reduction and social support enhancement.

There are also some limitations to our study. First, the study population came from a convenience sample of PLWH from Changsha CDC and may not represent PLWH from other clinics in other areas of China, extrapolation of the conclusion of this study should be particularly cautious. Second, the assessment of depression was based on a screening tool of PHQ-9 instead of a standard diagnostic tool. Third, our study has not comprehensively investigated all the factors that may influence depression and there may be additional factors that affect the model results such as PTG. Fourth, our study was focused on the impact of psychological factors on depression of PLWH, with much less attention paid to other equally important factors such as socio-demographic factors, which warrants further research. Fifth, there were more female and married participants in those who completed all three follow-ups than in those who did not, which may lead to an underestimation or overestimation of the risk of depressive symptoms among female and married participants. Finally, we investigated inconsistent time intervals during follow-up, which may lead to the loss of some important information, such as the continuous changes in the prevalence of depressive symptoms from the first year to the fifth year of diagnosis.

## 5. Conclusion

Our findings contribute to a better understanding of the association between HIV-related stress, social support, and depressive symptoms and shed light on the time effect. This study provides valuable theoretical and methodological insights for relevant health service providers. The results of this study suggest that HIV-related stress and social support predict depressive symptoms among PLWH over a five-year period. Our findings suggest that reducing HIV-related stress and improving social support in the early stages of diagnosis is extremely important in preventing and controlling depressive symptoms among PLWH.

## Data availability statement

The data analyzed in this study is subject to the following licenses/restrictions: The data that support the findings of this study are available on request from the corresponding author. The data are not publicly available due to privacy or ethical restrictions. Requests to access these datasets should be directed to DL, luodan_csu_2011@126.com.

## Ethics statement

The studies involving human participants were reviewed and approved by Ethics Committee, Xiangya School of Public Health, Central South University. The patients/participants provided their written informed consent to participate in this study.

## Author contributions

DL conceived and designed the cohort study, was responsible for study coordination and data management, and assisted in interpretation and manuscript writing. ZL analyzed data and wrote the first version of the manuscript. XC, JL, and ZX assisted in reviewing protocol and study coordination in the field and reviewed the manuscript. JL, ZX, and YH, critically reviewed the manuscript for important intellectual content. ZL, XC, JL, ZX, YH, and DL contributed to the acquisition, analysis, or interpretation of data. All authors contributed to the article and approved the submitted version.

## Funding

This study was supported by the National Natural Science Foundation of China (81202290) and the Natural Science Foundation of Hunan Province (2019 J40401). The funders played no role in the design of the study and collection, analysis, and interpretation of data and in writing the manuscript.

## Conflict of interest

The authors declare that the research was conducted in the absence of any commercial or financial relationships that could be construed as a potential conflict of interest.

## Publisher’s note

All claims expressed in this article are solely those of the authors and do not necessarily represent those of their affiliated organizations, or those of the publisher, the editors and the reviewers. Any product that may be evaluated in this article, or claim that may be made by its manufacturer, is not guaranteed or endorsed by the publisher.
