# Supplementary material for: HIV-related stress predicts depression over five years among people living with HIV
Source: Front Public Health. 2023 Jun 12;11:1163604. doi: 10.3389/fpubh.2023.1163604 (PMC10291293; doi:10.3389/fpubh.2023.1163604)
Supplement: Supplementary file 1 [file Table_1.docx]

Table S1 Interactions between support utilization and the three dimensions of stress

| Characteristics | Model 1β（95%CI） | ***P*** |
| --- | --- | --- |
| **Time** |  |  |
| T0 | 0.927（0.146，1.707） | 0.020 |
| T1 | -0.033（-0.636，0.636） | 1.000 |
| T2 |  |  |
| **Interaction** |  |  |
| Support utilization× Emotional stress | 0.128（0.113，0.143） | ＜0.001 |
| Support utilization × Social stress | -0.007（-0.016，0.002） | 0.123 |
| Support utilization × Instrumental stress | 0.024（0.008，0.040） | 0.004 |
